# Lithium-induced apoptotic cell death is not accompanied by a noticeable inflammatory response in the kidney

**DOI:** 10.3389/fphys.2024.1399396

**Published:** 2024-08-14

**Authors:** Irina Baranovskaya, Kevin Volk, Sati Alexander, Justine Abais-Battad, Mykola Mamenko

**Affiliations:** Department of Physiology, Medical College of Georgia, Augusta University, Augusta, GA, United States

**Keywords:** anti-inflammatory, apoptosis, lithium, nephrogenic diabetes insipidus, renal damage, macrophage polarization

## Abstract

Lithium (Li^+^) therapy is a valuable tool in psychiatric practice that remains underutilized due to safety concerns. Excessive plasma Li^+^ levels are nephrotoxic and can trigger a local immune response. Our understanding of the immunomodulatory effects of Li^+^ in the kidney is fragmentary. Here, we studied how immune mechanisms contribute to the development of Li^+^-induced adverse effects in the kidneys of C57BL/6NJ mice placed on a 0.3% lithium carbonate diet for 28 days. We combined histochemical techniques, immunoblotting, flow cytometry, qPCR and proteome profiler arrays to characterize renal tissue damage, infiltrating immune cells and cytokine markers, activation of pyroptotic and apoptotic cascades in the kidneys of mice receiving Li^+^-containing and regular diets. We found that biomarkers of tubular damage, kidney injury marker, KIM-1, and neutrophil gelatinase-associated lipocalin, NGAL, were elevated in the renal tissue of Li^+^-treated mice when compared to controls. This correlated with increased interstitial fibrosis in Li^+^-treated mice. Administration of Li^+^ did not activate the pro-inflammatory NLRP3 inflammasome cascade but promoted apoptosis in the renal tissue. The TUNEL-positive signal and levels of pro-apoptotic proteins, Bax, cleaved caspase-3, and caspase-8, were elevated in the kidneys of Li^+^-treated mice. We observed a significantly higher abundance of CD93, CCL21, and fractalkine, accumulation of F4.80^+^ macrophages with reduced M1/M2 polarization ratio and decreased CD4^+^ levels in the renal tissue of Li^+^-treated mice when compared to controls. Therefore, after 28 days of treatment, Li^+^-induced insult to the kidney manifests in facilitated apoptotic cell death without an evident pro-inflammatory response.

## Introduction

Lithium (Li^+^) stands out as one of the most effective tools in the psychiatric pharmacological arsenal, particularly in the treatment of bipolar disorder. Randomized clinical trials and real-world evidence studies establish Li^+^ as a mood stabilizer for bipolar patients with unique disease-modifying benefits including well-documented effective control of acute mania, prevention of manic as well as depressive episodes, improvement of the long-term disease trajectory, reduction of suicide rates and all-cause mortality (Malhi and Bauer, 2023; Kessing, 2024). Li^+^ can also improve cognition, reduce the incidence of dementia and has a promising therapeutic potential to combat Alzheimer’s, Parkinson’s and Huntington’s disease, as well as addiction disorders (Grandjean and Aubry, 2009; Singh et al., 2023).

Wider use of Li^+^ has been limited due to its perceived toxicity manifesting only at excessively high plasma levels (Rybakowski, 2018). Li^+^ has a narrow therapeutic window of 0.6–1.2 mM in plasma (Malhi and Bauer, 2023; Kessing, 2024), and chronic exposure to high doses of Li^+^ has been linked to a range of kidney pathologies. By far, the most common renal complication of Li^+^ therapy is impairment of vasopressin-dependent water reabsorption in the collecting duct principal cells leading to nephrogenic diabetes insipidus (NDI) that is observed in up to 40% of treated patients (Grunfeld and Rossier, 2009). Several studies report that Li^+^ can induce distal renal tubular acidosis, manifesting in increased urine pH and ammonia excretion, possibly due to altered proton and ammonia secretion by the collecting duct intercalated cells (Perez et al., 1975; Weiner et al., 2014). The prevalence of severe renal complications in Li^+^-treated population is relatively low (Vestergaard et al., 1979; Bendz et al., 2010). Infrequent observations of proximal tubular atrophy, progressive interstitial fibrosis, nephrotic syndrome, formation of microcysts, chronic kidney disease and renal failure have been reported in a small percentage of long-term Li^+^ users (Markowitz et al., 2000; Kripalani et al., 2009; Azab et al., 2015; Alsady et al., 2016; Rybakowski, 2018; Mehta et al., 2022). On the other hand, a mounting body of evidence demonstrates protective effects of Li^+^ in the kidney (Gong et al., 2016). Long-term therapy with low doses of Li^+^ as a monotherapy or in combination with other drugs demonstrated renoprotective effects in animal models of acute kidney injury and can be beneficial in chronic kidney disease (Bao et al., 2015; Alsady et al., 2016; Mehta et al., 2022; Shimizu et al., 2023). A better understanding of the mechanisms underlying Li^+^-induced adverse effects will allow mitigating the risks and ensure broader utilization and better outcomes of Li^+^-based therapies.

Li^+^-induced insult to the kidney can induce a local immune response. The accrued data draws a complex and often contradictory picture of the immunomodulatory action of lithium in the kidney. Intoxication observed at high plasma Li^+^ concentrations activates proinflammatory factors in the kidney and induces pyroptosis (Jing et al., 2022). At the same time, long-term administration of Li^+^-containing diet, resulting in therapeutically acceptable circulating Li^+^ levels, reportedly induces interstitial fibrosis in rat kidneys but is associated with minimal inflammatory activity (Walker et al., 2013). Other studies reveal anti-inflammatory properties of Li^+^ that can affect renal function. Lithium has been shown to inhibit the synthesis of prostaglandins, reduce renal injury and attenuate inflammation in several models of acute kidney injury and renal disease (Nahman et al., 2012; Azab et al., 2015; Gong et al., 2016). This creates a strong momentum for further evaluation of Li^+^-induced immune mechanisms in the kidney.

Based on the previous studies reporting insidious progression of Li^+^-induced renal injury, we hypothesized that Li^+^-induced insult to the kidney is not accompanied by a pronounced inflammation in the renal tissue. In this study, we placed C57BL/6NJ mice on a regular rodent chow containing 0.3% lithium carbonate for 4 weeks to achieve therapeutically acceptable serum Li^+^ levels and establish the contribution of immune mechanisms to nephrotoxic effects of lithium in the kidney. We demonstrate that administration of Li^+^ for 28 days induces NDI and moderate renal injury, likely due to apoptosis of tubular epithelial cells. The immune response localized to the kidneys of Li^+^-treated mice does not exhibit a distinctive proinflammatory phenotype and has characteristic anti-inflammatory features.

## Materials and methods

### Experimental animals

7-week-old male C57BL/6NJ mice (Jackson Laboratories, United States) weighing between 20–22 g were randomly assigned to receive Teklad 2,918 chow (control group) and Teklad 2,918 chow supplemented with 0.3% lithium carbonate (Li^+^-treated group) over the course of 28 days. All animal procedures were approved by the Institutional Animal Care and Use Committee of the Medical College of Georgia at Augusta University (AUP 2017-0844) and were conducted under the National Institutes of Health Guide for the Care and Use of Laboratory Animals. All mice had unrestricted access to water and food, were maintained under conditions of a 12:12 h light-dark cycle, constant temperature, and humidity. Animal wellbeing was monitored daily throughout the study. Body weight was recorded twice a week. At the end of each experimental week, the animals were transferred to metabolic cages (Tecniplast, Italy) for a 24-h period with *ad libitum* access to water and food. Urine volume, osmolarity, and the amount of consumed water were measured to assess NDI manifestations. At the end of the study, all animals were euthanized by exsanguination under 5% isoflurane anesthesia. Blood samples were collected through cardiac puncture into BD Vacutainer serum separation tubes to measure serum ion concentrations using the CareLyte Plus electrolyte analyzer (Diamond Diagnostics) or into BD Vacutainer blood collection tubes with sodium heparin for blood urea nitrogen (BUN) assessment with the STAT Profile Prime Plus Gas Analyzer (Nova Biomedical). Renal tissue was dissected and used for immune cell extraction, fixed for histological analysis, or rapidly frozen in liquid nitrogen and stored at −80°C for further molecular biology analysis.

### Histopathological staining

Fresh renal tissue was fixed in 10% neutral-buffered formalin for 24 h, embedded into paraffin, cut into 5 µm sections, mounted on slides, and stained with picrosirius red according to a routinely used histological protocol. Slides were scanned using Aperio Versa (40x) and digital images were downloaded into QuPath platform (version 0.5.0) (Bankhead et al., 2017). Interstitial fibrosis, interstitial thickness, and tubular dilation were analyzed in the cortex in a blinded manner (N = 5 per each animal group), Briefly, by loading a threshold classifier we identified the tissue as an area with the signal above the background (to exclude tubular lumen, arteries, and any technical voids in the tissue). This approach was used for all further analysis, including fibrosis and interstitial thickness. Tubular dilation was determined in the cortex by measuring the area with the signal equal to the background, manually excluding arteries, glomeruli, and any tissue-free space other than tubular lumen from consideration. Then, an object classifier was created and trained by the provided RTrees algorithm to detect the interstitial regions of the tissue area, which was further applied to calculate the interstitial area in the cortex (named interstitial thickness). Finally, by creating an additional threshold classifier in the red channel we calculated the percentage of interstitial fibrosis normalized on the whole cortex area (% of cortex) or the cortical interstitial area (% of interstitium).

### Evaluation of DNA fragmentation in formalin fixed paraffin embedded kidney sections

To evaluate cell apoptosis in the renal tissue, DNA fragmentation was visualized in the kidney sections using a terminal deoxynucleotidyl transferase dUTP nick end labeling (TUNEL) assay with a 3, 3 -diaminobenzidine (DAB)–horseradish peroxidase (HRP) detection staining, TUNEL-HRP-DAB kit (Abcam), according to the manufacturer’s guidelines. Briefly, 5 μm sections cut from the paraffin-embedded kidney blocks fixed in 10% formalin were deparaffinized, rehydrated and permeabilized with proteinase K for 20 min. Endogenous peroxidase activity was inhibited by a 3% H_2_O_2_ solution in methanol. This was followed by an application of TUNEL reaction mixture in a humidified chamber for 90 min, and a subsequent 10 min incubation in a blocking buffer. The sections were incubated with the streptavidin-HRP conjugate for 30 min, followed by an application of DAB for 10 min and methyl green counterstain for another 3 min. Quantification of the number of TUNEL-positive cells was manually performed in a blinded manner across the entire cortex area using an inverted Nikon Eclipse Ti2 microscope equipped with a 40x Nikon Plan Fluor Objective. The cortex area for each sample was calculated using the QuPath platform (version 0.5.0). The number of TUNEL-positive cells per square millimeter (mm^2^) in the cortex was used for statistical analysis. Representative images were taken with a Nikon DS-Fi3 digital camera.

### Western blotting

Renal tissue was homogenized in an ice-cold 5% sorbitol, 5 mM histidine/imidazole buffer (pH = 7.5) supplemented with a protease and phosphatase inhibitor cocktail (Thermo Scientific). Homogenate samples were further centrifugated at 2000 g for 10 min at +4°C and protein concentration was measured using standard Bradford assay protocol. Each sample was mixed with Laemmli sample buffer containing 5% β-mercaptoethanol and incubated for 5 min at +95°C. Then, samples were separated by SDS PAGE using 4%–15% precast polyacrylamide gel (Bio-Rad). Trans-Blot Turbo RTA Midi 0.2 µm nitrocellulose transfer kit (Bio-Rad) and Trans-Blot turbo transfer system (Bio-Rad) were used for protein transfer according to the manufacturer’s protocol. The membrane was stained with Ponceau red for protein load verification. Membranes were washed in Tris-buffered saline with 0.1% Tween-20 (TBST), blocked in 5% non-fat milk or in 5% BSA (for IL-1β and ASC detection) in TBST for 1 h at room temperature and further incubated with one of the primary antibodies for NGAL, KIM-1, Bax, Bcl-xL, Caspase-8, Cleaved Caspase-8, Cleaved Caspase-3, IL-1β, сaspase-1, сleaved сaspase-1, NLRP3, ASC, and Bid overnight at +4°C (Supplementary Table S1). The incubation with secondary horseradish peroxidase-conjugated antibody was performed for 1 h at room temperature. Stripping was performed when needed in a buffer containing 0.2 M Glycine, 3.5 mM SDS, and 1% Tween 20 (pH = 2.2) for 10 min. Information about the antibodies used and their working dilutions is provided in the Supplementary Material. Chemiluminescent SuperSignal Pico Plus kit and West Femto Maximum Sensitivity Substrate kits (Thermo Scientific), Azure C600 Imaging System (Azure Biosystems) and ChemiDoc (Bio-rad) were used for protein detection. Densitometric analysis was performed in ImageJ 1.50 software (NIH). The levels of specific protein bands were normalized to the total protein signal according to Ponceau red staining.

### Quantitative PCR analysis

Renal tissue was homogenized in TRIzol reagent (Invitrogen) using Fisherbrand 150 Handheld homogenizer (Fisher Scientific). RNA extraction and RNA samples digestion with DNAse (Promega) was followed by complementary DNA synthesis using Reverse Transcriptase Kit (Promega) and according to the manufacturer’s instructions. Quantitative PCR analysis was performed using Universal SYBR Green Supermix (Bio-Rad) and QuantStudio 3 RT-PCR System (Applied Biosystems). Primers were synthesized by Integrated DNA Technologies and provided in the Supplementary Table S1. The relative expression of Fn-1, Col1-a1, NLRP3, caspase-1, IL-1β and ASC genes in renal tissue homogenates was calculated by 2^−ΔΔCT^ method, and Rn18s/45s ribosomal RNA was used for normalization.

### Protein array analysis

Proteome Profiler Mouse XL Cytokine Array (R&D systems) was used for cytokine profile screening according to the manufacturer’s instructions. Renal tissue homogenate samples isolated from Li^+^-treated or control mice (N = 6 per group) were pooled together for each group and incubated on the nitrocellulose membranes containing capture antibodies printed in duplicates. Loading volume for each sample was adjusted using the Bradford assay. Dot blot densitometric analysis was performed in the ImageJ 1.50 software (NIH). To calculate the relative protein abundance, densities of all dots were normalized on the average density of the dots on the membrane with control group samples. Mean values of relative protein abundances were calculated for a pair of membranes containing samples from control and Li^+^-treated groups. Cytokine profiling was performed 4 times for each group.

### Immune cell isolation and analysis

Immune cell isolation from the renal tissue was performed using a well-established protocol as previously described (Walton et al., 2023). In brief, freshly isolated renal tissue was minced using a razor blade and incubated in the RPMI-1640 medium (Gibco) containing 0.1% collagenase type IV (Worthington) and 10 μg/mL DNAse I (Sigma-Aldrich). The incubation was carried out for 30 min at +37°C. To obtain single-cell suspension, kidney homogenates were sequentially filtered through a series of filters with pore sizes of 100, 70, and 40 µm. Subsequently, mononuclear cells were separated using Percoll density gradient centrifugation (Sigma-Aldrich). Isolated cells were counted using a hemocytometer. For further analysis, 1 million cells from each sample were incubated with antibodies against CD16/CD32 to block non-specific antibody binding. To identify specific immune cell populations, we used previously published panel of fluorochrome-conjugated antibodies (Saleh et al., 2016): CD45-BV510, F4/80-AlexFluor488, CD3-APC, CD4-APC/H7, and CD8a-PE/Cy7. Live and dead cells were distinguished using DAPI staining (1 μg/mL, BioLegend). Flow-cytometric analysis of the stained cells was performed using a 5-Laser AURORA Spectral Cytometer (Cytek). The gating strategy employed for the identification and analysis of immune cell populations is illustrated in Supplementary Figure S1.

### Macrophage extraction

Positive selection of the renal single-cell suspensions was performed by column separation using MicroBeads against F4.80 (Miltenyi Biotec) according to the manufacturer’s instructions. The purification quality was checked by flow cytometry and was more than 90%. RNA was extracted from samples using RNeasy Mini Kit (Qiagen). RNA samples were further used for RT-qPCR experiments, where the relative expression of IL-12β and Arg-1 mRNA was performed by 2^−ΔΔCT^ method (Supplementary Table S1).

### Statistical analysis

Statistical analysis and data visualization were performed in GraphPad Prism 9.41. Longitudinal data on urine osmolarity and volume, water consumption and body weight changes were analyzed by a two-way mixed-design ANOVA with a Greenhouse-Geisser correction. A Student’s t-test (with Welch’s correction for samples with unequal variances) was used to compare the endpoint data between control and Li^+^-treated groups. Values of P < 0.05 were considered statistically significant. Data are presented as mean ± SEM.

## Results

### Therapeutic serum Li^+^ levels cause a moderate insult to the renal tissue

**FIGURE 1:**
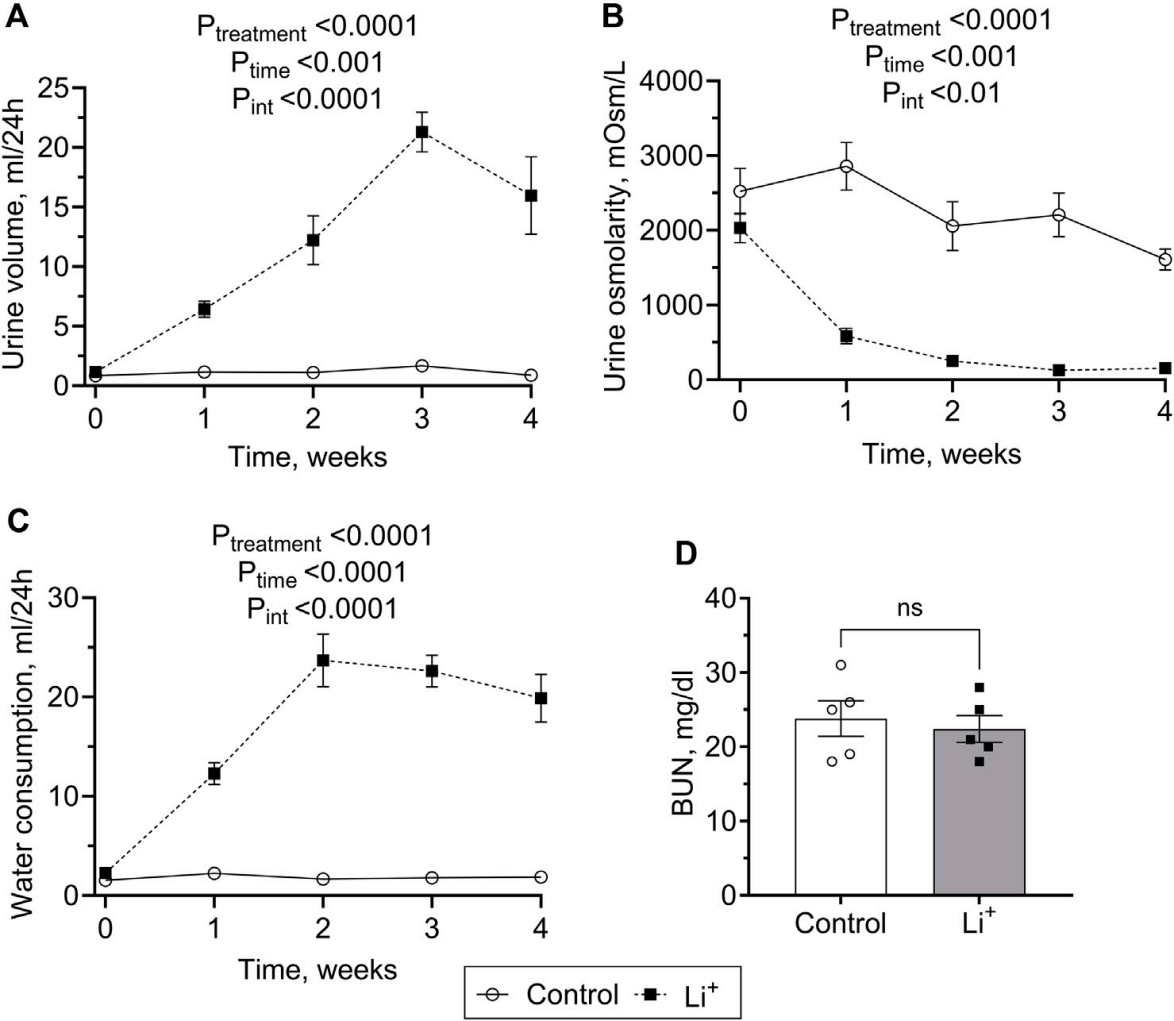
Lithium carbonate intake induces NDI symptoms in mice. Summary plots showing 24-h urine volume **(A)**, urine osmolarity **(B)** water consumption **(C)** measured over the course of 4 weeks and the endpoint BUN **(D)** observed in Li^+^-treated and control mice. Data are shown as mean ± SEM. Differences between group means were analyzed by a two-way mixed-design ANOVA with a Greenhouse-Geisser correction **(A–C)** and a Student’s t-test **(D)**. N ≥ 5 animals per group.

After 28 days on a 0.3% Li_2_CO_3_ diet, serum Li^+^ levels in mice were elevated to 0.7–1.5 mmol/L (Table 1). This is below the serum Li^+^ toxicity range (>1.5 mmol/L) reported for psychiatric patients (Grandjean and Aubry, 2009). Water intake, serum sodium and chloride concentrations, urine volume were significantly higher, and urine osmolarity was markedly lower in Li^+^-treated mice than in controls (Table 1; Figures 1A–C), strongly indicative of nephrogenic diabetes insipidus (NDI). Otherwise, Li^+^ intake did not appear to affect renal function, as indicated by similar endpoint BUN concentrations in Li^+^-treated and control mice (Figure 1D).

**TABLE 1:** Endpoint physiological parameters for experimental mice.

Parameter	Control		Li^+^-treated		Significance
	Mean	SEM	Mean	SEM	*p*-Value
Final body weight, g	27.67	0.96	21.47	0.48	**< 0.0001** ζ
Total kidney weight, mg	338.3	11.12	261.2	7.99	**< 0.0001**
Total kidney to body weight, %	1.22	0.02	1.22	0.03	0.82
Serum Li^+^, mmol/L	ND	-	1.34	0.23	
Serum Na^+^, mmol/L	148.7	0.31	153.0	1.50	**0.020** ζ
Serum K^+^, mmol/L	4.37	0.42	4.95	0.43	0.35
Serum Cl^−^, mmol/L	113.7	0.32	117.9	0.90	**0.001** ζ
BUN, mg/dL	23.80	2.40	22.40	1.81	0.65
Water intake, ml/24 h	1.86	0.12	19.88	2.40	**< 0.0001** ζ
Urinary volume, ml/24 h	0.89	0.02	15.96	3.26	**< 0.01** ζ
Urinary osmolality, mOsm/L	1,608.00	140.00	153.00	37.64	**< 0.01** ζ

A Student’s t-test (with Welch’s correction for samples with unequal variances–ζ) was used for statistical comparisons. Data are presented as mean ± SEM. *N* = 10 for each group. ND, not determined.

*P* values lower than 0.05 are indicated in bold.

**FIGURE 2:**
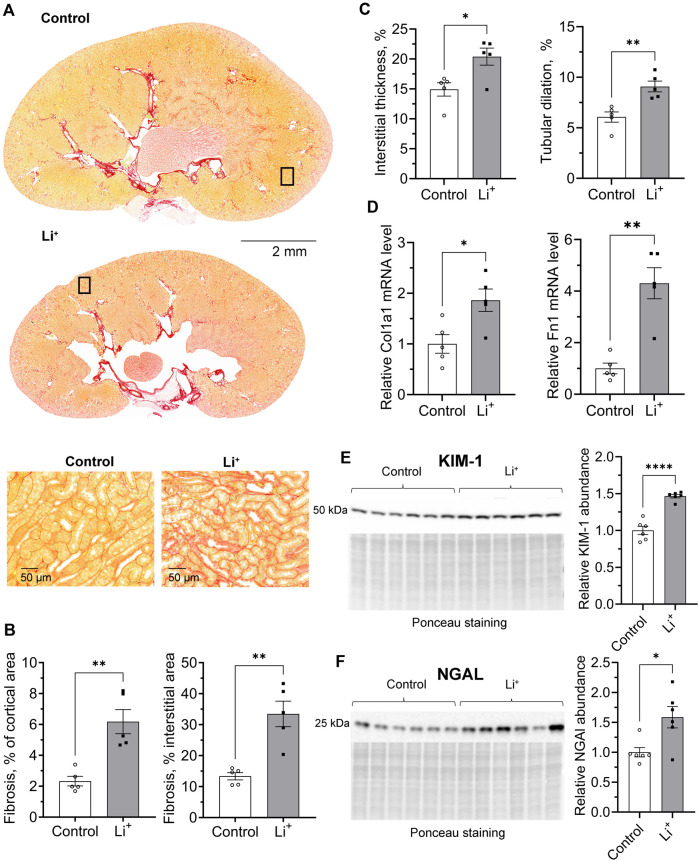
Chronic exposure to lithium causes a moderate insult to the renal tissue. **(A)** Representative microphotographs showing renal tissue fibrosis evaluated by the picrosirius red staining in control and Li^+^-treated mice. **(B)** Summary plots on the interstitial fibrosis that was calculated as the percentage of the fibrotic area in the cortex and cortical interstitium. **(C)** Graphs summarizing the quantitative analysis of the interstitial thickness and tubular dilation in the cortex. **(D)** Summary plots showing the relative expression levels of Collagen type I alpha 1 and Fibronectin 1 in renal homogenates by RT-qPCR. Graphs summarizing protein abundances of kidney injury markers, KIM-1 **(E)** and NGAL **(F)** detected by immunoblotting in renal tissue homogenates. Data are shown as mean ± SEM. Differences between groups were analyzed using a Student’s t-test with Welch’s correction for samples with unequal variances. * – p < 0.05, ** – p < 0.01, **** – p < 0.0001, N ≥ 5 animals per group.

Renal histology revealed morphological changes in the renal cortex including a higher percentage of fibrotic tissue, increased interstitial thickness, tubular dilation, and clear signs of hydronephrosis in renal papillae of Li^+^-treated mice compared to the control group (Figures 2A–C). Quantitative RT-PCR showed that the expression of genes encoding extracellular matrix proteins associated with increased interstitial thickness and fibrosis, collagen type I alpha 1 (Col1a1) and fibronectin 1 (Fn1), was significantly higher in renal tissue homogenates isolated from Li^+^-treated mice than in controls (Figure 2D). Protein abundance of the proximal and distal tubule cell damage markers, kidney injury molecule, KIM-1 (Figure 2E) and neutrophil gelatinase-associated lipocalin, NGAL (Figure 2F), were moderately but significantly elevated in the renal tissue of Li^+^-treated mice when compared to controls.

### Li^+^ changes the cytokine profile and alters the levels of lymphoid and myeloid cells in the renal tissue

**FIGURE 3:**
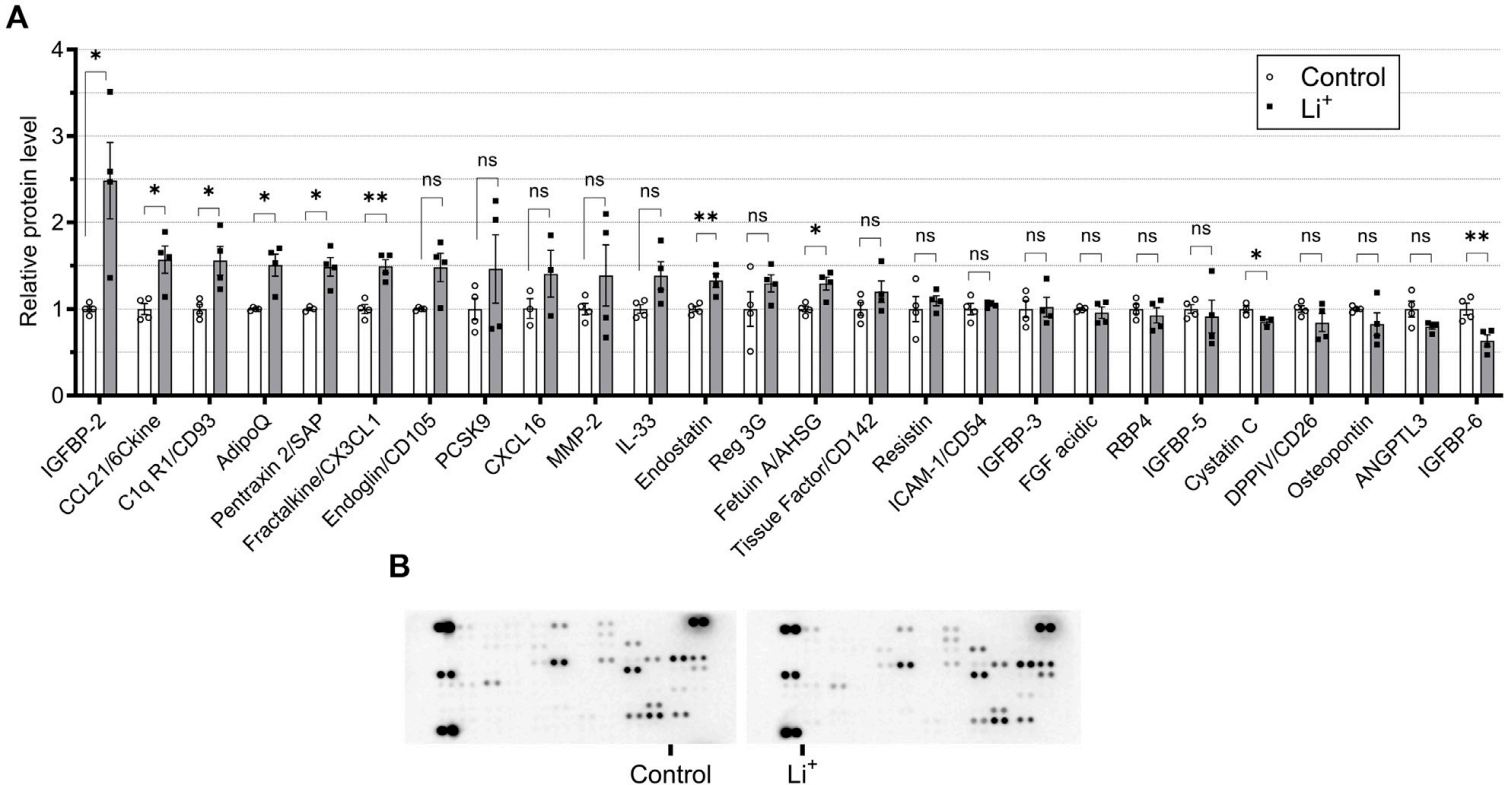
Administration of lithium does not trigger a pro-inflammatory response in the kidney. **(A)** A plot summarizing cytokine array data on the cytokines and chemokines in the renal tissue isolated from control and Li^+^-treated mice. **(B)** Representative images of dot-blot cytokine array membranes visualizing the reporting signal density from kidney homogenates of control and Li^+^-treated mice. Data are shown as mean ± SEM. Differences between groups were analyzed using a Student’s t-test with Welch’s correction for samples with unequal variances. * – p < 0.05, ** – p < 0.01. N = 6 mice per group. To perform the cytokine screening, renal tissue homogenates from all control or Li^+^-treated mice were pooled together and applied onto respective membranes. The procedure was repeated 4 times.

To investigate if Li^+^-induced damage is accompanied by an inflammatory response in the renal tissue, we performed cytokine and chemokine screening in the renal tissue samples from Li^+^-treated and control mice using a multiplex cytokine array. The abundance of anti-inflammatory markers, pentraxin 2, adiponectin and CD93, was higher in the renal tissue of Li^+^-treated mice than in controls. The levels of pro-inflammatory markers, CD26, CLCX16, PCSK9, and osteopontin, were not significantly different in the renal tissue samples isolated from Li^+^-treated and control mice. Interestingly, the abundance of CCL21/6Ckine and fractalkine/CX3CL1 was elevated in the kidneys of Li^+^-treated mice when compared to controls (Figures 3A, B).

**FIGURE 4:**
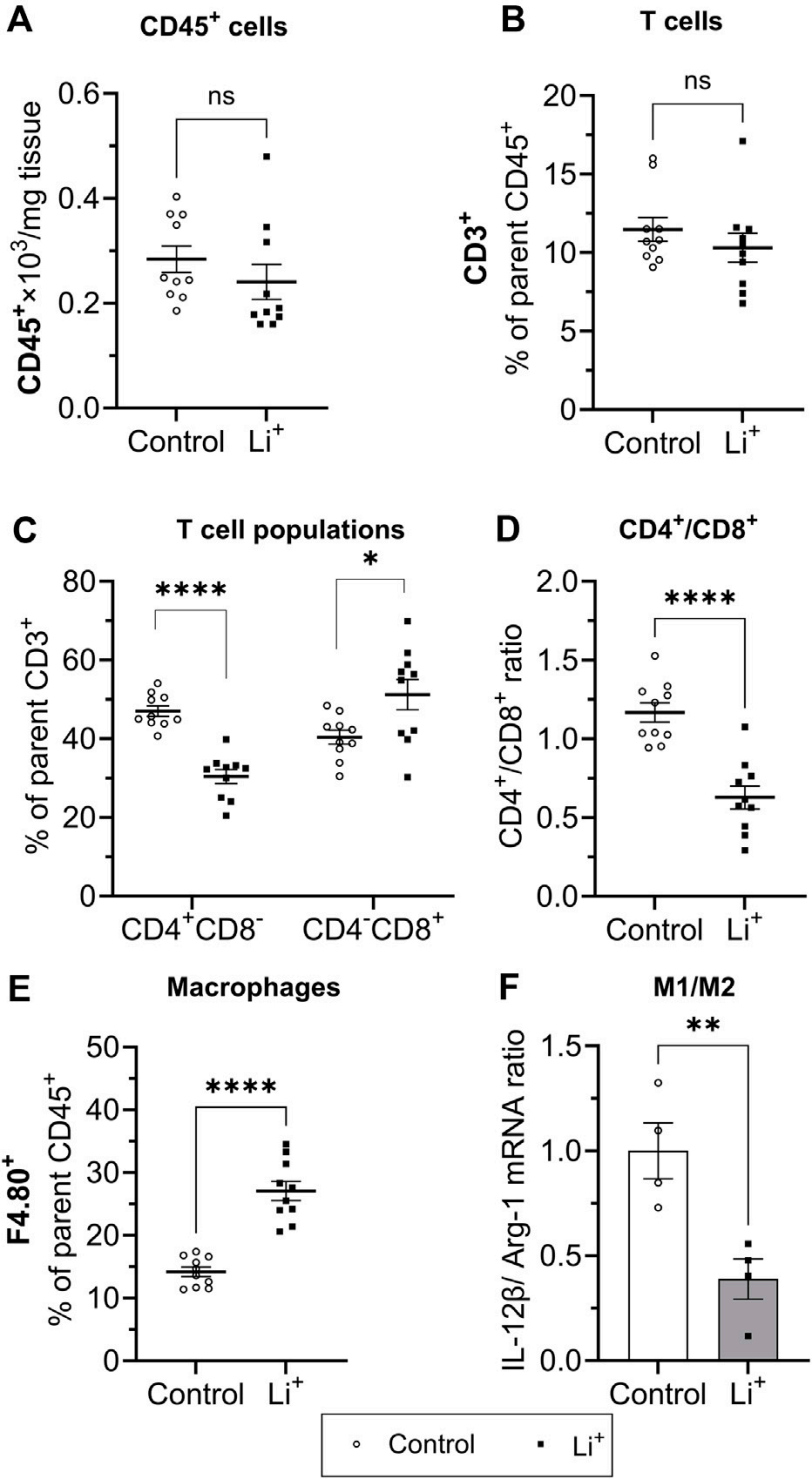
Lithium changes the immune cell landscape in the kidney. Summary plots demonstrating flow-cytometric analysis of immune cells isolated from the renal tissue: total lymphocytes [CD45^+^, **(A)**]; T lymphocyte [CD3^+^, **(B)**; helper (CD4^+^CD8^−^] and cytotoxic (CD4^−^CD8^+^) T lymphocytes **(C)**; the ratio of CD4^+^/CD8^+^ T lymphocytes **(D)**; macrophages [F4.80^+^, **(E)**]. **(F)** A summary graph showing the ratio of IL-12b/arginase-1 mRNA expression in a purified F4.80^+^ macrophage fraction. Data are shown as mean ± SEM. Differences between groups were analyzed using a Student’s t-test with Welch’s correction for samples with unequal variances. *p < 0.05, **p < 0.01, ****p < 0.0001, N ≥ 4 mice per group.

Next, we performed immunophenotyping of the renal tissue samples isolated from control and Li^+^-treated mice using flow cytometry. There were no detectable differences in the total number of immune cells (CD45^+^ per mg renal tissue) and in the percentage of T cells (CD3^+^, %) between the groups (Figures 4A, B). Interestingly, the percentage of helper T lymphocytes (CD4^+^CD8^−^, %) was significantly lower and the proportion of cytotoxic T lymphocytes (CD4^−^CD8^+^, %) – significantly higher in the kidneys of Li^+^-treated mice than in control (Figure 4D). The proportion of macrophages (F4.80^+^, %) was almost two-fold higher in the renal tissue of Li^+^-treated mice when compared to the control group (Figure 4C). Based on earlier reports (Orecchioni et al., 2019), we used the ratio of IL-12β/arginase-1 gene expression to assess macrophage polarization along the M1/M2 (pro-/anti-inflammatory) axis. The IL-12β/arginase-1 mRNA ratio was significantly lower in the renal tissue macrophages of Li^+^-treated mice (Figure 4E). This shift in macrophage polarization is consistent with a reduced inflammatory response in the kidneys of Li^+^-treated mice when compared to controls.

### Li^+^ activates apoptosis, but does not stimulate the pro-inflammatory NLRP3 inflammasome cascade in the kidney

**FIGURE 5:**
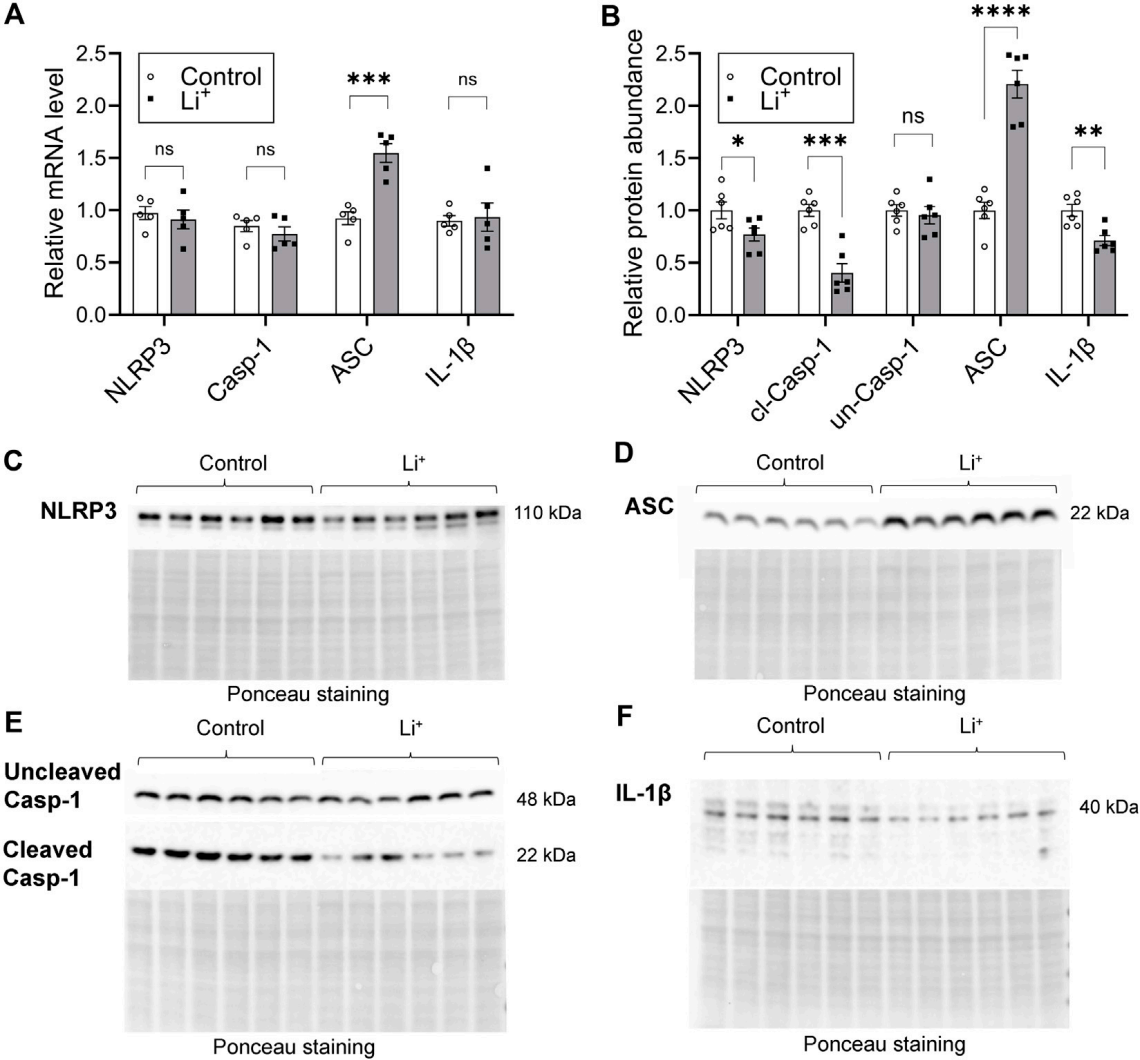
Lithium does not activate the NLRP3 inflammasome cascade in the kidney. **(A)** A summary plot demonstrating relative expression of inflammasome-associated genes: NLRP3, caspase 1, ASC, IL-1β in the renal tissue of control and Li^+^-treated mice. **(B)** A summary plot showing the abundance of NLRP3, uncleaved and cleaved caspase 1, ASC, IL-1β proteins in the kidney homogenates isolated from control and Li^+^-treated mice. **(C–F)** Representative image of membranes stained for NLRP3, uncleaved and cleaved caspase 1, ASC, IL-1β proteins. Total protein Ponceau staining is shown below the respective bands of interest. Data are shown as mean ± SEM. Differences between groups were analyzed using a Student’s t-test with Welch’s correction for samples with unequal variances. *p < 0.05, **p < 0.01, ***p < 0.001, ****p < 0.0001. N ≥ 5 mice per group.

Serum Li^+^ levels (>2.5 mmol/L) exceeding the toxicity threshold reportedly result in the activation of NLRP3 inflammasome triggering inflammatory cell death through pyroptosis in the mouse kidney (Jing et al., 2022). The priming stage of NLRP3 inflammasome activation results in NFκB-mediated transcriptional upregulation of the inflammasome components and substrates. Thus, next we assessed the expression of genes associated with NLRP3 inflammasome – interleukin-1β pathway, known to induce pyroptosis and the release of inflammatory cytokines. The mRNA levels of NLRP3, caspase-1, and IL-1β were similar in the renal tissue of Li^+^-treated and control mice (Figure 5A). Renal abundance of uncleaved caspase-1 protein was comparable in both mouse groups, while the protein levels of NLRP3, cleaved caspase-1 and IL-1β were significantly lower in the kidneys of Li^+^-treated mice when compared to controls (Figures 5B–F). Interestingly, the expression of apoptosis-associated speck-like protein containing a caspase activation and recruitment domain (ASC) gene and its protein abundance was markedly higher in the renal tissue isolated from Li^+^-treated mice when compared to controls (Figures 5A, B, D). Importantly, while ASC is required for inflammasome activation, it also has a pronounced apoptotic function and can induce apoptosis in a p53-dependent manner (Protti and De Monte, 2020).

**FIGURE 6:**
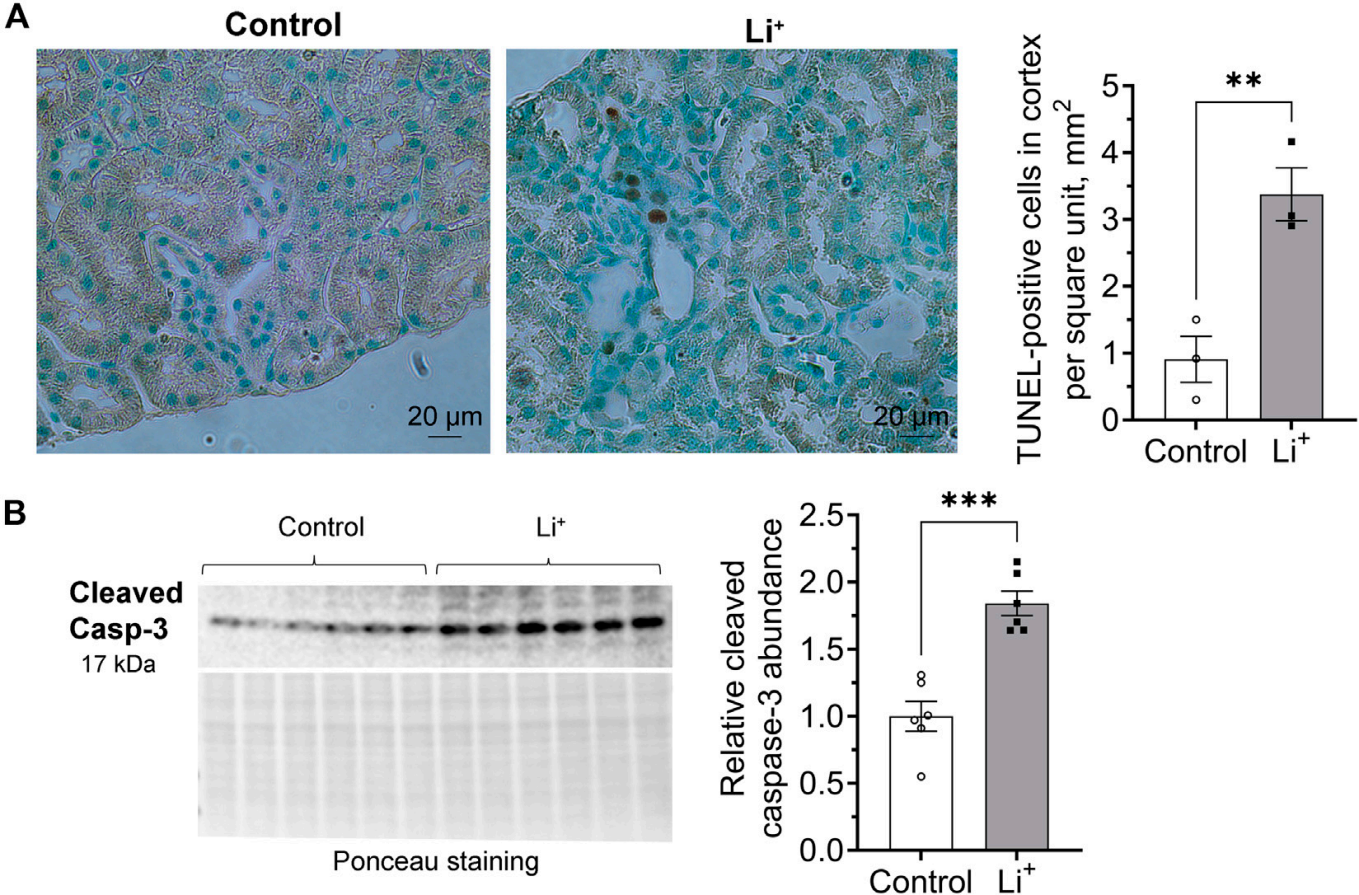
Lithium induces apoptosis in the renal tissue. **(A)** Representative images of kidney sections isolated from control and Li^+^-treated mice stained with an HRP/DAB TUNEL assay kit (left). A graph summarizing the analysis of TUNEL-staining data (right). **(B)** A representative image of the membrane stained for cleaved caspase-3 and total protein (Ponceau) and a summary graph showing the abundance of cleaved caspase-3 in the renal tissue homogenates isolated from control and Li^+^-treated mice. Data are shown as mean ± SEM. Differences between groups were analyzed using a Student’s t-test with Welch’s correction for samples with unequal variances. **p < 0.01, ***p < 0.001. N ≥ 3 mice per group.

We used TUNEL assay to examine DNA fragmentation as an indicator of apoptotic cell death in the renal tissue. The number of TUNEL-positive cells was significantly higher in the renal cortex of Li^+^-treated mice than in controls (Figure 6A). Since apoptotic, necrotic and even proliferating cells with increased rates of DNA repair may exhibit TUNEL-positive staining, we further compared the abundance of apoptotic markers in the renal tissue of Li^+^-treated and control mice with immunoblotting. Signaling cascades that induce apoptotic cell death converge on the cleavage and activation of caspase-3 that plays a major role in the executive phase of apoptosis. We found that cleaved caspase-3 was significantly more abundant in the kidney homogenates isolated from Li^+^-treated mice than in controls, corroborating our TUNEL staining data on the activation of apoptosis in the renal tissue of Li^+^-treated mice (Figure 6B). Thus, Li^+^ induces apoptotic cell death in the kidney, but does not activate NLRP3 inflammasome mediated pyroptosis.

### Li^+^ triggers apoptosis in the kidney through both extrinsic and mitochondrial pathways

**FIGURE 7:**
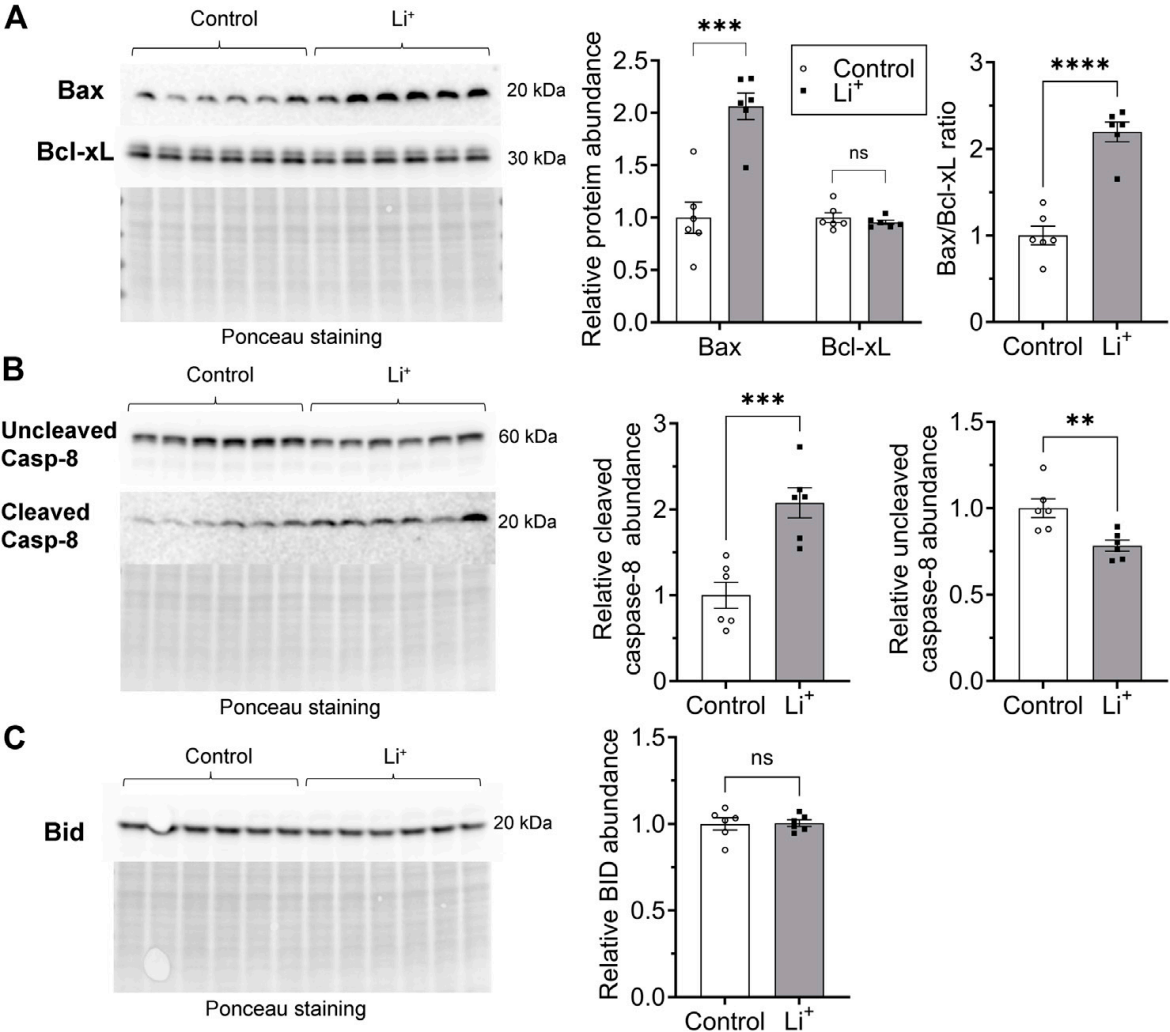
Lithium induces apoptosis in renal cells via extrinsic and intrinsic pathways. **(A)** Representative images of the membranes and summary graphs showing the abundance of Bax and Bcl-xL in the renal tissue homogenates isolated from control and Li^+^-treated mice. **(B)** Representative images of the membranes and summary graphs showing the abundance of full and cleaved caspase-8 in the renal tissue of control and Li^+^-treated mice. **(C)** A representative image of the membrane and a summary plot demonstrating the abundance of Bid protein in the kidneys of control and Li^+^-treated mice. Images of the membranes stained with Ponceau to control protein loading are presented for each experiment. Data are shown as mean ± SEM. Differences between groups were analyzed using a Student’s t-test with Welch’s correction for samples with unequal variances. **p < 0.01, ***p < 0.001, ****p < 0.0001, N ≥ 5 mice per group.

Apoptosis can be induced through intrinsic and extrinsic pathways. The intrinsic pathway activation relies on a mitochondrion-centered interaction between pro-apoptotic and anti-apoptotic proteins from Bcl-2 family, such as Bax and Bcl-xL, that either promotes apoptotic cell death or generates a pro-survival signal. We found that the abundance of the pro-apoptotic Bax protein is significantly higher in the renal tissue from Li^+^-treated mice than in controls, while the level of the pro-survival Bcl-xL protein is similar in both groups (Figure 7A). The extrinsic receptor-mediated apoptotic pathway induces cleavage and activation of caspase-8. The abundance of cleaved caspase-8 was two times higher in the renal tissue from Li^+^-treated mice than in controls, while the levels of uncleaved pro-caspase-8 were significantly lower in the kidneys of Li^+^-treated mice (Figure 7B). In addition to its central role in the extrinsic apoptotic pathway, caspase-8 can activate the BH3 interacting-domain death agonist, Bid (Kantari and Walczak, 2011). The activation of Bid, a Bcl-2 protein family member, amplifies the caspase-8 mediated pro-apoptotic signal and links the extrinsic receptor-mediated and intrinsic mitochondria-driven apoptotic pathways. We observed similar levels of Bid protein in the renal tissue samples isolated from Li^+^-treated and control mice (Figure 7C). Therefore, chronic lithium intake independently activates receptor-mediated and mitochondrial apoptotic pathways in the renal cells.

## Discussion

In this study, C57BL/6NJ male mice were placed on a 0.3% lithium carbonate diet for 28 days to achieve serum lithium concentrations below the reported toxicity range for psychiatric patients and explore chronic effects of Li^+^ on the kidney. This intervention led to the development of NDI, renal tissue fibrosis, and elevated levels of kidney injury markers, KIM-1 and NGAL. Li^+^ administration did not result in activation of the NLRP3 inflammasome pathway in the renal tissue but promoted apoptosis in tubular cells. Protein abundance of CD93, CCL21, and fractalkine, important for efficient macrophage infiltration, was significantly higher in the kidneys of Li^+^-treated mice when compared to controls. And, indeed, we observed markedly elevated levels of F4.80^+^ macrophages in the renal tissue of Li^+^-treated mice with a polarization shift towards M2 phenotype. The abundance of anti-inflammatory markers, including pentraxin 2 and adiponectin, was increased in the kidneys of mice receiving Li^+^ when compared to controls, while pro-inflammatory cytokines remained mostly unaffected. Consistent with the anti-inflammatory profile of Li^+^-induced immune response in the kidney, the levels of pro-inflammatory proteins, associated with NLRP3-inflammasome activation, caspase-1, NLRP3, and IL-1β, were lower in the renal tissue of Li^+^-treated mice. While a 4-week treatment with Li^+^ did not elevate the levels of immune cells (CD45^+^) in the renal tissue, significant alterations were found in the T cell population balance. The numbers of CD4^+^ cells were significantly lower and the levels of CD8^+^ cells were markedly elevated in the kidneys of Li^+^-treated mice when compared to controls.

We show that after 28 days on 0.3% Li_2_CO_3_ diet mice attained serum Li^+^ concentrations in the range of 0.7–1.5 mmol/L, and their kidneys exhibited clear signs of damage and fibrosis (Figure 2). Long-term exposure to lithium has been associated with tubular atrophy and interstitial fibrosis in human and rodent models (Walker et al., 2013; Alsady et al., 2016). The accrued evidence suggests the involvement of glycogen synthase kinase-3 beta (GSK-3β), a negative regulator of Wnt/β-catenin activity (Huang et al., 2023). Prolonged activation of the Wnt-pathway can exacerbate fibrotic kidney diseases and accelerate the progression of chronic kidney disease (CKD) (Xiao et al., 2016). Lithium, as a GSK-3β inhibitor, can over-activate the Wnt/β-catenin pathway, thereby promoting the development of renal fibrosis (Alsady et al., 2016). Higher levels of KIM-1 and NGAL markers in the kidneys of Li^+^-treated mice corresponded to the renal damage occurring in the proximal and distal tubular segments, respectively. Excessive accumulation of Li^+^ in these nephron segments has been linked to proximal tubular atrophy, dysregulation of AQP2 trafficking and expression, and loss of principal collecting duct cells (Alsady et al., 2016). Thus, the dietary intervention and experimental timeline used in our study are reflective of chronic exposure to Li^+^ in psychiatric patients and animal models.

Comparison of the cytokine and immune cell profiles in the renal tissue samples from Li^+^-treated and control mice strongly suggests the absence of an evident proinflammatory response after 28 days on Li^+^ diet. Administration of Li^+^ did not significantly change the abundance of pro-inflammatory markers, such as CD26, CXCL16, PCSK9, and osteopontin in the kidney. At the same time the levels of anti-inflammatory markers, including pentraxin 2 and adiponectin, were significantly elevated in the renal tissue of Li^+^-treated mice compared to control animals (Figure 3A). The levels of CD45^+^ remain comparable in both groups, while the number of CD4^+^ T cells, associated with inflammation in multiple renal disorders, including salt-sensitive hypertension (Walton et al., 2023), was significantly lower in the renal tissue of Li^+^-treated mice when compared to controls (Figure 4). We also show that the abundance of a pro-inflammatory protein CD26, expressed on the activated CD4^+^ T cells under Th1 polarization (Ohnuma et al., 2008), was comparable in the renal homogenates isolated from Li^+^-treated and control mice (Figure 3A). Moreover, the ratio of IL-12β/arginase-1 gene expression, reflecting the proinflammatory macrophage polarization along the M1/M2 axis, was significantly lower in the renal tissue macrophages of Li^+^-treated mice when compared to controls (Figure 4F). Finally, the protein abundance of cleaved caspase-1, critical for initiation of an inflammatory response, was remarkably lower in the renal tissue of Li^+^-treated mice compared to controls (Figure 5E). Earlier studies demonstrate that treatment with Li^+^ reduces the levels of pro-inflammatory cytokines in the blood of bipolar patients (Boufidou et al., 2004) and decreases neuroinflammation in rats (Basselin et al., 2010). An animal study shows that long-term administration of Li^+^- containing diet over the course of 6 months induces apoptosis in the epithelium of dilated and atrophic cortical tubules in rat kidneys but is accompanied with minimal inflammatory activity (Walker et al., 2013). Thus, our observations are consistent with previously reported anti-inflammatory properties of lithium.

Intoxication caused by excessively high levels of Li^+^ (>2.5 mmol/L) in serum and renal tissue has been associated with an inflammatory reaction in mouse kidney, including the activation of NLRP3 inflammasome and pyroptosis (Jing et al., 2022). At serum Li^+^ levels below 1.5 mmol/L in our experimental cohort, we did not observe elevated transcription of NLRP3 inflammasome-related genes or increased abundance of respective protein products in the renal tissue of Li^+^-treated mice (Figure 5). This is in line with our findings pointing to the anti-inflammatory nature of Li^+^-induced immune response in the kidney. One notable exception was significantly higher expression of ASC in the kidneys of Li^+^-treated mice compared to controls (Figures 5A, B, D). While ASC is critical for inflammasome-mediated cell death through pyroptosis, it also plays an essential role in the intrinsic mitochondrial pathway of apoptosis through the p53-Bax network (Protti and De Monte, 2020). We confirmed the activation of apoptosis in the renal tissue of Li^+^-treated mice. The number of tubular epithelial cells with DNA fragmentations characteristic of apoptosis was significantly elevated in the renal cortex of Li^+^-treated mice (Figure 6A). Cleavage of the effector caspase-3 (Figure 6B), associated with activation of apoptosis, and Bax/Bcl-xL ratio (Figure 7A), indicative of mitochondrial apoptotic pathway activation, were markedly higher in the kidneys of Li^+^-treated mice when compared controls. The observed elevated levels of cytotoxic (CD8^+^) T lymphocytes in the renal tissue of Li^+^-treated mice indicate that Li^+^ may also rely on the extrinsic pathway to induce apoptosis (Diamond and Gill, 2000). Indeed, we saw higher levels of cleaved caspase-8, an initiator caspase in receptor-mediated apoptotic cell death, in the kidneys of mice receiving Li^+^ (Figure 7B). Caspase-8 activated through the extrinsic pathway can cleave Bid protein that gets translocated into mitochondria, where it promotes Bax activation, linking the extrinsic receptor-mediated and intrinsic mitochondria-dependent pathways (Kantari and Walczak, 2011). This linkage amplifies the apoptotic receptor-mediated apoptotic signal by engaging the mitochondrial pathway. However, Bid levels were comparable in the renal tissue of Li^+^-treated and control mice (Figure 7C), indicative of independent activation of mitochondrial and extrinsic pathways by Li^+^ administration. Thus, we conclude that chronic exposure to lithium likely contributes to the induction of apoptosis in the kidney through both mitochondrial and extrinsic pathways, without appreciable involvement of inflammasome or pyroptosis.

We found significantly elevated levels of CD93, fractalkine/CX3CL1, CCL21, and IGFBP-2 in the renal tissue samples isolated from Li^+^-treated mice (Figure 3A). CD93 glycoprotein is a phagocytic receptor required for efficient clearance of apoptotic cells by macrophages *in vivo* (Norsworthy et al., 2004). CX3CR1, a receptor for fractalkine, reportedly contributes to CKD progression (Cormican and Griffin, 2021). However, it can also promote macrophage trafficking to apoptotic sites, the release of anti-inflammatory mediators, such as interleukin-1 receptor antagonist and prostaglandin E2 (Zhuang et al., 2017). CX3CR1 deficiency was previously associated with a shift toward M1 dominant macrophages in the liver (Ni et al., 2022). The role of IGFBP-2 in renal pathophysiology and modulation of immune response remains to be fully understood. Proinflammatory and proapoptotic effects were reported in diabetic kidney disease (Wang et al., 2024), while in cancer IGFBP2 can induce M2 macrophage polarization (Zhang et al., 2024). Finally, CCL21 and its receptor CCR7 also facilitate the accumulation of macrophages and CD45^+^ type I collagen+ fibroblasts into the renal tissue (Tang et al., 2019). It was shown that anti-CCL21 therapy successfully suppresses the infiltration of bone-marrow-derived fibroblasts (CD45^+^/ColI^+^) and reduces renal fibrosis (Sakai et al., 2006). In our study, renal tissue F4.80^+^ macrophages were almost 2 times more abundant in Li^+^-treated mice than in controls and exhibited polarization toward M2 type. M2 polarization of macrophages is known to be profibrotic in the kidney (Feng et al., 2018) and could potentially contribute to the development of renal fibrosis, a known pathology of chronic lithium treatment. This provides a strong rationale for further research investigating molecular determinants of Li^+^-induced immune response in the renal tissue.

Overall, we demonstrate that administration of lithium for 28 days, resulting in circulating Li^+^ levels below the clinically reported toxic threshold, causes an insidious insult to the kidney. Li^+^ promotes apoptosis in tubular cells but does not induce pronounced inflammation in the renal tissue. On the contrary, Li^+^-induced immune response in the kidney exhibits distinct anti-inflammatory features. Future experiments assessing the molecular determinants of apoptotic cell death and characterizing the subsets of lymphoid and myeloid cells in the renal tissue and at the systemic level will be critical to better understand the beneficial effects and pathophysiological sequelae of chronic lithium administration on the kidney.

## Data Availability

The raw data supporting the conclusions of this article will be made available by the authors, without undue reservation.
