# Supplementary material for: Lithium-induced apoptotic cell death is not accompanied by a noticeable inflammatory response in the kidney
Source: Front Physiol. 2024 Aug 14;15:1399396. doi: 10.3389/fphys.2024.1399396 (PMC11373137; doi:10.3389/fphys.2024.1399396)
Supplement: Supplementary file 1 [file DataSheet1.docx]

Supplementary Material

Lithium-induced apoptotic cell death is not accompanied by a noticeable inflammatory response in the kidney

Irina Baranovskaya*, Kevin Volk, Sati Alexander, Justine Abais-Battad, Mykola Mamenko*

*** Correspondence:** Mykola Mamenko: mmamenko@augusta.edu

Irina Baranovskaya: ibaranovskaya@augusta.edu

## Supplementary Figure


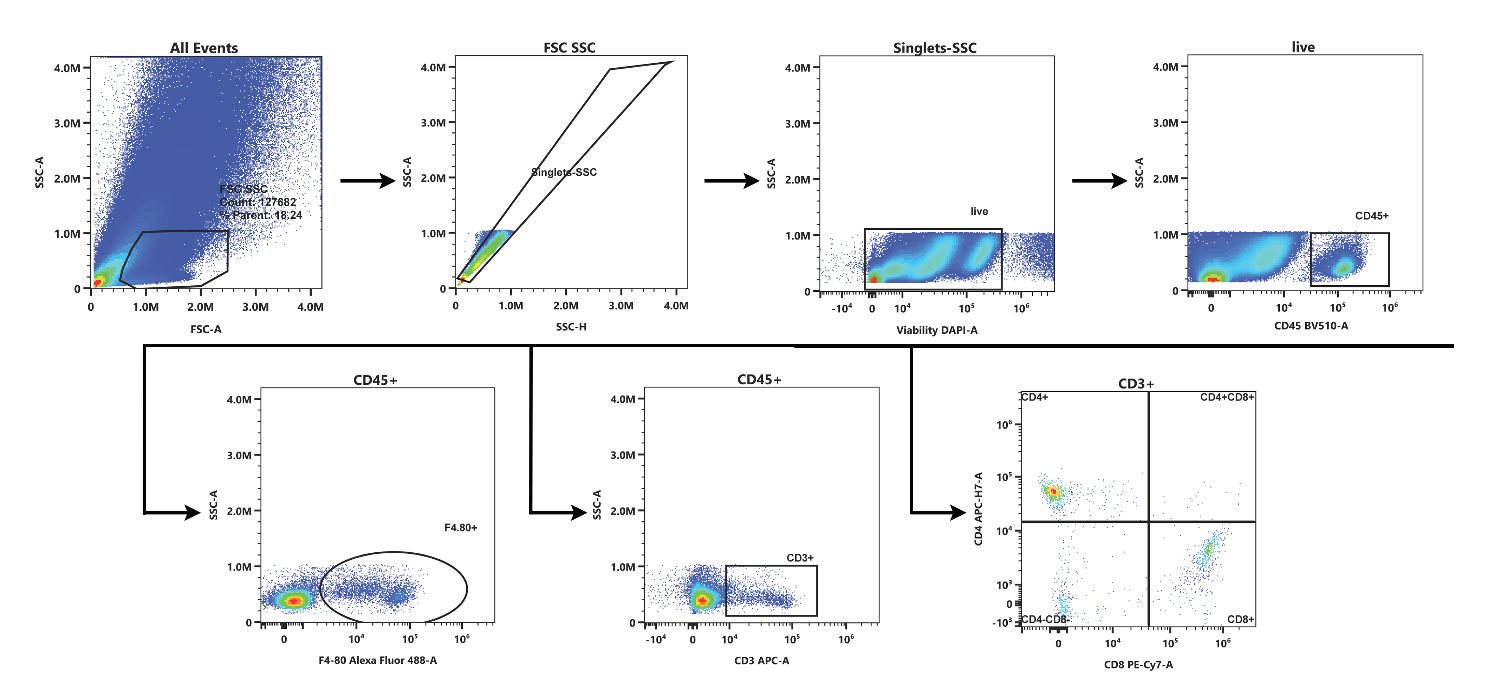


**Figure S1. Representative gating strategy.**

# Table. Major Resources.

**Primers** were used for real-time PCR analysis in this study. Source: Integrated DNA Technologies.

| **Gene** | **Primer Sequence (5'->3')** | **Sample type** |
| --- | --- | --- |
| IL-1β | CAAATCTCGCAGCAGCACATCAAC | RNA isolated from kidney homogenates |
|  | GGGAACGTCACACACCAGCAG |  |
| Casp-1 | GTATCCAGGAGGGAATATGTG |  |
|  | ACAACACCACTCCTTGTTTCTC |  |
| NLRP3 | TGGTGACCCTCTGTGAGGTG |  |
|  | TCTTCCTGGAGCGCTTCTAA |  |
| ASC | AGACATGGGCTTACAGGA |  |
|  | CTCCCTCATCTTGTCTTGG |  |
| Rn18s/45s ribosomal RNA | CTCAACACGGGAAACCTCAC |  |
|  | CGCTCCACCAACTAAGAACG |  |
| Fn-1 | CCCTATCTCTGATACCGTTGTCC |  |
|  | TGCCGCAACTACTGTGATTCGG |  |
| Col1-a1 | CCTCAGGGTATTGCTGGACAAC |  |
|  | CAGAAGGACCTTGTTTGCCAGG |  |
| IL-12β | TGGTTTGCCATCGTTTTGCTG | RNA isolated from F4.80^+^ macrophages |
|  | ACAGGTGAGGTTCACTGTTTCT |  |
| Arg-1 | AGACAGCAGAGGAGGTGAAGAGTAC |  |
|  | GGTAGTCAGTCCCTGGCTTATGGT |  |

**Antibodies**

| **Name** | **Source** | **Catalog #** | **Dilution factor** | **Method** |
| --- | --- | --- | --- | --- |
| NGAL Polyclonal Antibody | Invitrogen | PA5-46938 | 1:200 | Immuno-blotting |
| KIM-1 Polyclonal Antibody | Novus Biologicals | NBP1-76701 | 1:1000 |  |
| Bax Rabbit Monoclonal Antibody | Cell Signaling | 14796 | 1:1000 |  |
| Bcl-xL Rabbit Monoclonal antibody | Cell Signaling | 2764 | 1:1000 |  |
| Cleaved Caspase-3 (Asp175) Rabbit Monoclonal Antibody | Cell Signaling | 9661S | 1:1000 |  |
| Cleaved Caspase-8 (Asp387) Polyclonal Antibody | Cell Signaling | 9429 | 1:1000 |  |
| Caspase-8 (D35G2) Rabbit Monoclonal Antibody | Cell Signaling | 4790 | 1:1000 |  |
| BID Rat Monoclonal Antibody | R&D Systems | MAB860SP | 1:500 |  |
| IL-1β (D6D6T) Rabbit Monoclonal Antibody | Cell Signaling | 31202 | 1:1000 |  |
| NLRP3 Rabbit Polyclonal Antibody | Abclonal | A12694 | 1:2000 |  |
| Cleaved Caspase-1 (Asp296) (E2G2I) Rabbit Monoclonal Antibody | Cell Signaling | 89332 | 1:1000 |  |
| Caspase-1 (E2Z1C) Rabbit Monoclonal Antibody | Cell Signaling | 24232 | 1:1000 |  |
| ASC/TMS1 (D2W8U) Rabbit Monoclonal Antibody | Cell Signaling | 67824 | 1:1000 |  |
| Donkey Anti-Rat IgG H&L (HRP) | Abcam | ab102182 | 1:10 000 |  |
| Horseradish peroxidase-conjugated Antibody | Jackson ImmunoResearch Laboratories | 111035144 | 1:10 000 |  |
| CD16/CD32 | BD Biosciences | 553142 | 1:200 | Flow Cytometry |
| CD45-BV510 | BioLegend | 103138 | 1:200 |  |
| AlexFluor488-F4/80 | BioLegend | 123120 | 1:200 |  |
| CD3-APC | BD Biosciences | 553066 | 1:200 |  |
| CD4-APC/H7 | BD Biosciences | 560181 | 1:200 |  |
| CD8a-PE/Cy7 | BioLegend | 100722 | 1:200 |  |

**Chemicals, media**

| **Description** | **Source** | **Catalog #** | **Method** |
| --- | --- | --- | --- |
| TUNEL Assay Kit - HRP-DAB | Abcam | ab206386 | Tunel |
| Limonene Mounting Medium | Abcam | ab104141 |  |
| Protease and phosphatase inhibitor cocktail | Thermo Scientific | A32959 | Immuno-blotting |
| 4–15% precast polyacrylamide gel | Bio-Rad | 5678085 |  |
| Trans-Blot Turbo RTA Midi 0.2 µm Nitrocellulose Transfer Kit | Bio-Rad | 1704271 |  |
| Non-Fat Dry Milk | Lab Scientific | M0841 |  |
| SuperSignal West Pico PLUS Chemiluminescent Substrate | Thermo Scientific | 34577 |  |
| SuperSignal West Femto Maximum Sensitivity Chemiluminescent Substrate | Thermo Scientific | 34094 |  |
| Trizol Reagent | Invitrogen | 15596018 | qRT-PCR |
| RNeasy Mini Kit | Qiagen | 74104 |  |
| RQ1 RNase-Free DNase | Promega | M6101 |  |
| Reverse Transcriptase System | Promega | A3500 |  |
| iTaq Universal SYBR Green Supermix | Bio-Rad | 1725121 |  |
| RPMI-1640 | Gibco | 22400-089 | Flow Cytometry |
| Collagenase type IV | Worthington | CLS-4 |  |
| DNAse I | Sigma-Aldrich | D5025 |  |
| Percoll | Sigma-Aldrich | P1644-100ML |  |
| Falcon cell strainers 100, 70, 40μm | Falcon | 352360, 352340, 352350 |  |
| DAPI | BioLegend | 422801 |  |
| Proteome Profiler Mouse XL Cytokine Array | R&D systems | ARY028 | Protein array |
| Anti-F4/80 MicroBeads UltraPure, mouse | Miltenyi Biotec | 130-110-443 | Macrophage polarization (qRT-PCR) |
| LS Columns | Miltenyi Biotec | 130-042-401 |  |
